# Quality of life reported by survivors after hospitalization for Middle East respiratory syndrome (MERS)

**DOI:** 10.1186/s12955-019-1165-2

**Published:** 2019-06-11

**Authors:** Sarah Batawi, Nehal Tarazan, Rajaa Al-Raddadi, Eman Al Qasim, Anees Sindi, Sameera AL Johni, Fahad M. Al-Hameed, Yaseen M. Arabi, Timothy M. Uyeki, Basem M. Alraddadi

**Affiliations:** 1 King Abdulaziz University, Jeddah, Saudi Arabia; 2 King Faisal Specialist Hospital and Research Center, Jeddah, Saudi Arabia; 3 Department of Community Medicine, King Abdulaziz University, Jeddah , Saudi Arabia; 4 King Abdullah International Medical Research Center, King Abdulaziz Medical City – National Guard Health Affairs, Riyadh, Saudi Arabia; 5 King Saud Bin Abdulaziz University for Health Sciences, Riyadh, Saudi Arabia; 6 King Abdullah International Medical Research Center, King Saud bin Abdulaziz University for Health Sciences, Intensive Care Department, King Abdulaziz Medical City – National Guard Health Affairs, Jeddah , Saudi Arabia; 7 Influenza Division, National Center for Immunization and Respiratory Diseases, Centers for Disease Control and Prevention, Atlanta, Georgia USA; 8 Department of Medicine, King Faisal Specialist Hospital and Research Center, University of Jeddah, P.O BOX 40047 Jeddah 21499 MBC J 46, Jeddah, Saudi Arabia

**Keywords:** Health-related quality of life (HRQoL), Coronavirus, Middle East respiratory syndrome (MERS), Pneumonia, Saudi Arabia, Severe acute respiratory infection (SARI), Long term outcome, survivors

## Abstract

**Introduction:**

Data are lacking on impact of Middle East Respiratory Syndrome (MERS) on health-related quality of life (HRQoL) among survivors.

**Methods:**

We conducted a cross-sectional survey of MERS survivors who required hospitalization in Saudi Arabia during 2016–2017, approximately 1 year after diagnosis. The Short-Form General Health Survey 36 (SF-36) was administered by telephone interview to assess 8 quality of life domains for MERS survivors and a sample of survivors of severe acute respiratory infection (SARI) without MERS. We compared mean SF-36 scores of MERS and non-MERS SARI survivors using independent t-test, and compared categorical variables using chi-square test. Adjusted analyses were performed using multiple linear regression.

**Results:**

Of 355 MERS survivors, 83 were eligible and 78 agreed to participate. MERS survivors were younger than non-MERS SARI survivors (mean ± SD): (44.9 years ±12.9) vs (50.0 years ±13.6), *p* = 0.031. Intensive care unit (ICU) admissions were similar for MERS and non-MERS SARI survivors (46.2% vs. 57.1%), *p* = 0.20. After adjusting for potential confounders, there were no significant differences between MERS and non-MERS SARI survivors in physical component or mental component summary scores. MERS ICU survivors scored lower than MERS survivors not admitted to an ICU for physical function (*p* = 0.05), general health (*p* = 0.01), vitality (*p* = 0.03), emotional role (*p* = 0.03) and physical component summary (*p* < 0.02).

**Conclusions:**

Functional scores were similar for MERS and non-MERS SARI survivors. However, MERS survivors of critical illness reported lower quality of life than survivors of less severe illness. Efforts are needed to address the long-term medical and psychological needs of MERS survivors.

## Introduction

Middle East Respiratory Syndrome (MERS) caused by a novel coronavirus (MERS-CoV) was first identified in Saudi Arabia in 2012 [1]. Since then, MERS outbreaks have occurred in several hospitals [2, 3]. As of March 31, 2019, 2399 laboratory-confirmed MERS cases, including 827 deaths (34.5%) worldwide have been reported to the World Health Organization (WHO [4]. The clinical presentation of MERS is variable, ranging from asymptomatic infection to critical illness and multi-organ failure requiring intensive care unit (ICU) admission for mechanical ventilation, renal replacement therapy, and vasopressor support for refractory shock [5–8]. A recent post-mortem study reported detection of MERS-CoV by transmission electron microscopy in the lungs, kidney, and muscle of a fatal case [9]. Lung pathology of a previous fatal case of MERS demonstrated diffuse alveolar damage without evidence of extrapulmonary spread [10]. MERS-CoV infection triggers specific CD4 and CD8 T-cell responses that may last up to 18–34 months following the primary infection [11, 12]. MERS severity and recovery has been correlated with CD4 T-cell response, levels of antibody and longevity, but not CD8 T-cell responses [11–15].

Patients who survived severe acute respiratory infection (SARI) due to non-MERS etiologies have reported persistent abnormalities during prospective follow up [16]. A longitudinal 2-year follow-up of survivors of severe illness from avian influenza A(H7N9) virus infection reported residual impairment in ventilation and diffusion capacity for carbon monoxide (DLCO) in more than half despite significant improvement from baseline during the first 6 months [17]. Survivors of a closely related coronavirus infection, Severe Acute Respiratory Syndrome (SARS), had residual abnormalities detected with pulmonary function testing, with impairment in DLCO up to 2 years after recovery [18]. In addition to air space changes reported in chest radiographs, further pulmonary abnormalities were observed using high resolution computed tomography [19] and impairment in Health-related quality of life (HRQoL) was also reported in SARS survivors [18].

While the clinical course and the immunological response to MERS-CoV infection have been described, data are lacking on the impact of MERS on quality of life among survivors. We aimed to describe the long-term outcomes and quality of life among survivors of MERS who required hospitalization. In addition, we compared reported HRQoL scores between MERS patients who had severe disease and those with mild disease.

## Methods

### Study design and setting

During February 1, 2016 to February 14, 2017, we aimed to evaluate MERS patients reported to the Ministry of Health (MOH) of Saudi Arabia from September 2014 until November 16, 2015 and who survived. Eligible patients met the following criteria: 1) had laboratory confirmed MERS [MERS-CoV RNA was detected in upper or lower respiratory tract specimens by real-time reverse transcription polymerase chain reaction (rRT-PCR)] [20]; 2) aged ≥18 years; 3) were hospitalized for MERS; 4) survived for at least 1 year from the time of MERS diagnosis; and 5) had contact information available to the MOH. A sample of SARI patients who tested negative for MERS-CoV RNA in upper or lower respiratory tract specimens by rRT-PCR (non-MERS SARI) was selected to serve as a comparative cohort. We identified potential non-MERS-SARI controls randomly from retrospective records, based on the WHO case definition of SARI; 1) Any acute respiratory infection requires hospitalization with history of fever or measured temperature ≥ 38 °C; 2) cough; 3) onset within the last 10 days [21]; and 4) aged ≥18 years. Disease severity was stratified based on requirements of admission to an intensive care unit or medical ward.

We excluded 1) subjects with incomplete information on quality of life survey and 2) patients who were not able to complete the interview. Non-MERS SARI cases were selected only from one hospital; whereas the MERS cases were from multiple hospitals in Saudi Arabia.

Contact information and the data registry of MERS-CoV patients were provided by the MOH in Saudi Arabia and the Ministry of National Guard Health Affairs Hospitals (Jeddah and Riyadh) and a short form health survey was administered through telephone interview by a general physician. Verbal informed consent was obtained from all participants.

### Short form health survey (SF-36)

The SF- 36 is an internationally recognized instrument that has been used in clinical trials to assess the quality of life for patients with other respiratory infections, including those caused by avian influenza A(H7N9) virus (17), influenza A(H1N1)pdm09 virus [22], and SARS-CoV [18]. The SF-36 consists of 36 question that evaluates eight health domains: physical functioning (PF), social functioning (SF), role limitation due to physical problems (RP), role limitation due to emotional problems (RE), mental health (MH), bodily pain (BP), vitality (VT), and general health (GH) [23, 24]. These domains can be further represented as physical (PSC) and mental (MCS) component summary scales. Scores for each domain can range from 0 (worst) to 100 (best) with higher scores indicating better HRQoL [24]. The Arabic version of SF-36 has been validated in the Saudi Arabian population, and no significant mean differences between Arabic and English SF-36 questionnaires were observed [25]. Patients were interviewed using the English or Arabic version as needed of the Short-Form 36-item survey (SF-36) to assess the HRQoL.

### Statistical analysis

Categorical data were expressed as frequencies and percentages, continuous data were reported as means ± SD for normally distributed data and medians and interquartile range (IQR) for non-normally distributed data. For comparisons of mean SF-36 scores between MERS and non-MERS SARI survivors, we used the independent t-test. Categorical variables were compared using the chi-square test. Multiple linear regression was used to adjust for the following potential confounders: Age, gender, ICU admission, healthcare worker, mean time of diagnosis to interview, presence of at least one comorbidity; and immunocompromised status.

We included age and gender as covariates since these factors have been shown to affect the HRQoL [26, 27]. Other covariates were included because these variables were significantly different (*p* < 0.05) between MERS vs non-MERS SARI patients. Analyses were performed using SPSS. *P*-values of < 0.05 were considered statistically significant.

### Ethics statement

Institutional Review Board approval was obtained from the Ministry of Health and the Ministry of National Guard Health Affairs of Saudi Arabia.

## Results

### Baseline characteristics of MERS and non-MERS SARI survivors

**Fig. 1:**
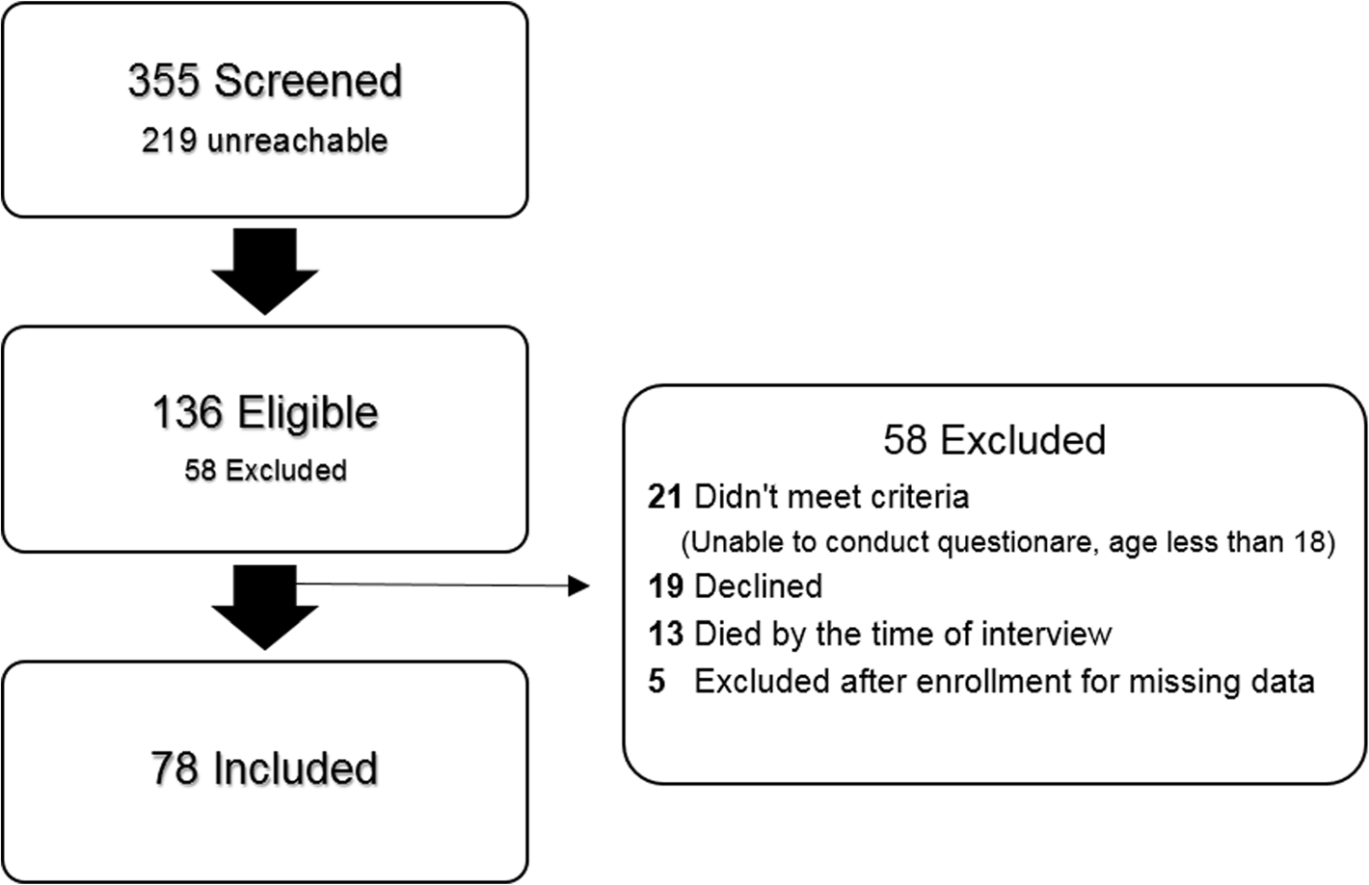
**a** MERS survivor recruitment process

Throughout the recruitment period from February 1, 2016 to February 142,017, 83 MERS survivors were determined to be eligible and 78 agreed to participate and were included in the final analysis (Fig. 1a). Eligible patients were interviewed from four major cities in the Kingdom: Riyadh 64 (82%), Jeddah 9 (12%), Ahsa 4 (5%) and Taif 1 (1%). Fifty-seven non-MERS SARI survivors met our inclusion criteria and were included in the analysis. MERS survivors were younger when compared to non-MERS SARI survivors (mean ± SD): (44.9 years ±12.9) vs (50.0 years ±13.6), *p* = 0.031.. The mean time from illness onset to interview was significantly longer in the non-MERS SARI cases than MERS survivors, mean (±SD), 25.3 months (7.5) and 13.8 months (3.4) respectively (*p* < 0.01). Twenty (25.6%) MERS survivors were healthcare personnel, whereas only two (3.5%) of the non-MERS survivors worked in a hospital at the time of illness onset (p < 0.01). Non-MERS survivors were more likely than MERS survivors to report a pre-existing medical condition. (Table 1).

**Table 1:** Baseline Characteristics of patients with Middle East Respiratory Syndrome (MERS) and patients with Non-MERS Severe Acute Respiratory Infection (SARI) at the time of diagnosis

Characteristic	MERS *N* = 78 (%)	Non-MERS SARI *N* = 57 (%)	*p*-value
Age-years (Mean ± SD)	(44.99 ± 12.95)	(50.04 ± 13.64)	0.31
Male gender	56 (71.8%)	34 (59.6%)	0.14
Nationality-Saudi	56 (71.8%)	34 (59.6%)	0.14
ICU admission	36 (46.2%)	32 (57.1%)	0.20
Mechanically ventilated	26 (33.3%)^c^	26 (45.6%)	0.10
Health care worker	20 (25.6%)	2 (3.5%)	< 0.01
Presence of comorbidity (one or more)	45 (63.4%)	44 (91.7%)	< 0.01
Comorbidities			
Diabetes	29 (37.2%)	28 (50.9%)	0.12
Hypertension	29 (37.2%)	29 (51.8%)	0.09
Respiratory disease	2 (2.6%)	0	0.33
Cardiovascular disease	10 (12.8%)	19 (33.9%)	< 0.01
Neurological disease	4 (5.1%)	12 (21.4%)	< 0.01
Renal disease	9 (11.5%)	15 (26.8%)	0.02
Hemodialysis	3 (3.9%)	3 (5.4%)	0.70
Chronic liver disease	0	7 (12.5%)	< 0.01
Immunocompromised^a^	6 (7.7%)	20 (35.7%)	< 0.01
Current smoker or history of smoking	26 (33.3%)	11 (19.6%)	0.08
Time to interview after illness –month (Mean ± SD)	(13.79 ± 3.43)	(25.32 ± 7.54)	< 0.01
Returned to original work at the time of interview	44 (88%)^b^	17 (73.9%)	0.17

^a^Immunocompromised patients were defined as follows: (Use of systemic corticosteroids or immunosuppressive medication, preexisting organ transplantation and active cancer)

^b^Subjects who were originally not working were excluded in the analysis

^c^Percentage from total patients with MERS (78) and Non-MERS SARI (57)

### Main outcomes

MERS survivors (*n* = 78) had significantly higher mean SF-36 scores than non-MERS SARI patients (*n* = 57) for the following domains (Table 2): physical functioning [mean ± SD (72.47 ± 26.11) vs (55.26 ± 35.07), p < 0.01], physical role [(64.87 ± 39.12) vs (50.00 ± 42.52), *p* = 0.04], general health [(73.03 ± 22.68) vs (63.95 ± 23.52), *p* = 0.03], vitality [(65.96 ± 26.47) vs (53.60 ± 28.80), *p* = 0.01] and physical component summary (71.33 ± 22.20) vs (59.77 ± 27.00), *p* = 0.01]. After adjusting for potential confounders, there were no significant differences between the physical or mental component summary scores between the two groups At the time of the survey, the majority of MERS survivors (88%) had returned to their original work compared with 74% of non-MERS SARI survivors, *p* = 0.17.

**Table 2:** Average score of SF36 components reported by MERS survivors (*n* = 78) and non-MERS SARI survivors (*n* = 57)

SF36 component	MERS (*n* = 78) mean (SD)	Non-MERS SARI survivors (*n* = 57) mean (SD)	*p*-value
Physical functioning	72.47 (26.11)	55.26 (35.07)	< 0.01
Physical role	64.87 (39.12)	50.00 (42.52)	0.04
Pain	78.97 (29.84)	76.05 (33.88)	0.60
General health	73.03 (22.68)	63.95 (23.52)	0.03
Vitality	65.96 (26.47)	53.60 (28.80)	0.01
Social functioning	84.45 (24.36)	75.44 (32.04)	0.08
Emotional role	75.12 (36.39)	62.58 (42.76)	0.08
Mental health	79.64 (22.34)	74.74 (24.03)	0.23
Physical component Summary	71.33 (22.20)	59.77 (27.00)	0.01
Mental component Summary	79.77 (23.20)	70.92 (28.53)	0.06

Mean SF-36 scores reported among MERS survivors stratified by disease severity are summarized in Table 3. SF-36 domain scores for those admitted to an intensive care unit (*n* = 36) were significantly lower than survivors managed in medical wards (*n* = 42) in the following domains, physical functioning [mean ± SD (66.94 ± 30.29) vs (78.69 ± 18.78), *p* < 0.05], general health [(65.94 ± 26.97) vs (79.10 ± 16.21), *p* = 0.01], vitality [(58.47 ± 31.60) vs (72.38 ± 19.29), *p* = 0.03], emotional role [(65.74 ± 38.62) vs (83.33 ± 33.13), *p* = 0.03], and physical component summary [(64.84 ± 25.52) vs (76.90 ± 17.35), *p* = 0.02] (Table 3). After adjustment for potential confounders, MERS ICU survivors had a significantly lower mental component summary score than MERS survivors cared for on medical wards (*p* < 0.026), but no significant difference was found in physical component summary score.

**Table 3:** Average score of SF36 components reported by MERS-CoV survivors admitted to an ICU (*n* = 36) or a medical ward (non ICU) (*n* = 42)

	MERS ICU (*n* = 36) mean (SD)	MERS Non ICU (*n* = 42) mean (SD)	*P* value
Physical functioning	66.94 (30.29)	78.69 (18.78)	0.05
Physical role	58.33 (40.53)	72.02 (36.29)	0.12
Pain	74.51 (33.55)	82.29 (26.34)	0.27
General health	65.94 (26.97)	79.10 (16.21)	0.01
Vitality	58.47 (31.60)	72.38 (19.29)	0.03
Social functioning	81.60 (28.74)	86.89 (19.90)	0.36
Emotional role	65.74 (38.62)	83.33 (33.13)	0.03
Mental health	77.11 (24.46)	81.81 (20.40)	0.36
Physical component Summary	64.84 (25.52)	76.90 (17.35)	0.02
Mental component Summary	74.82 (25.14)	84.01 (20.77)	0.09

Continuous data were compared using independent t-test

## Discussion

In this study, the overall quality of life reported by MERS survivors at approximately 14 months of follow-up was lower than that reported by non-MERS SARI survivors, although the difference became insignificant when adjusted for relevant confounders. In addition, survivors who required intensive care unit admission reported significantly lower overall quality of life than MERS survivors with less severe illness who were hospitalized in general medical wards. MERS survivors also reported significantly lower quality physical health at approximately 14 months after illness onset compared to a previously published sample of healthy individuals in Saudi Arabia.

Our findings are similar to those reported in studies of long-term follow-up of survivors of SARI due to other etiologies associated with high mortality. The overall scores of SF-36 domains (with exception for mental health and role emotion) were significantly lower in survivors of SARS-CoV at 2 years compared with the general Hong Kong population [18]. Similar findings were observed at 1-year follow-up evaluation among patients who survived critical illness and the acute respiratory distress syndrome from influenza A(H1N1)pdm09 virus infection compared with a sample of the general population [22]. In a recent study that assessed survivors of severe disease due to avian influenza A(H7N9) virus infection in China, quality of life reported at approximately 1.5 years was lower than a sample of the general population [17].

Longitudinal studies have documented some improvements in quality of life reported over time [28]. However, the changes are not uniformly distributed across the SF36 components. In SARS-CoV survivors, the quality-of-life scores for the following domains (social functioning, role physical and role emotional) were lowest at discharge, increased substantially at 1 year [29], and remained stable at 2 years [18]. In another SARS-CoV survivor study, there was a significant improvements in role physical, social function, and role emotional domains at one-year evaluation [16, 17]. However, a study of survivors who were hospitalized with avian influenza A(H7N9) virus infection did not find any significant changes in reported SF-36 domains from 3 to 24 months post-discharge follow-up [17].

MERS survivors had a similar degree of impairment identified on HRQoL compared to SARI survivors of non-MERS-CoV origin. Despite the longer interval in the assessments in non-MERS SARI survivors, reported quality of life scores were similar after adjustments of potential confounders using a linear regression model. Due to the descriptive cross-sectional study design, any temporal relationship may be undetected; MERS could have a transient effect on survivors at discharge, and it is possible we were unable to detect these dynamic changes. Corticosteroid-induced myopathy, muscle wasting and weakness have been reported in survivors of ARDS at one-year follow up [30]. Since many MERS ICU patients have been treated with high-dose corticosteroids, sequelae from such therapy could contribute to reduced quality of life in survivors. Additionally, critically ill MERS survivors had impairment in the emotional role that might be attributed to psychological trauma similar to that observed in survivors of the 2003 SARS outbreak [31].

Our study is subject to several limitations. First, selection bias may be present; as the small study population may not be representative of all eligible Saudi MERS survivors -since the majority of survivors were unreachable- and our non-MERS SARI controls were enrolled from only two hospitals in Saudi Arabia. Additionally, the number of non-MERS SARI controls was lower than MERS survivors. Second, we had limited medical information on potential predictors that may alter the reported quality of life scores. Specifically, we did not have information regarding the severity of illness such as APACHE II score, number of ventilator days, length of hospital stay, or use of sedatives or neuromuscular blocking agents which have been shown to affect long term outcomes. Third, there is a potential for recall bias since there was a significant difference in time from illness onset to interview time between MERS cases and non-MERS SARI controls; to reduce bias, we performed a multiple linear regression model to adjust for confounders. Lastly, we administered the SF-36 questionnaire by telephone and not in-person. However, telephone administration has been validated in previous studies and shown to be a reliable method when compared to a self-administered survey [32, 33].

This cross-sectional observational study has provided preliminary information about the quality of life of MERS survivors. Assessment of functional limitation and exercise capacity is needed in MERS survivors to estimate the long-term burden of this illness. Prospective longitudinal studies measuring objective parameters such as; pulmonary function testing, 6-min walk test and detection of depression, anxiety and post-traumatic stress disorder with correlation with health status, will provide more informative data to understanding of the overall long term outcomes of MERS-CoV infection.

## Conclusions

Approximately 14 months after onset of MERS illness, ICU survivors reported higher limitations in some measure of their quality of life than patients with less severe illness who were managed in medical wards. The long-term consequences of MERS illness on survivors may be similar to that caused by SARI of other etiologies. Further attention is needed to address the long-term medical and psychological needs of survivors of MERS and non-MERS SARI.

## Data Availability

The datasets used and/or analysed during the current study are available from the corresponding author on reasonable request.

## Abbreviations

BP: Bodily pain
DLCO: Diffusion capacity for carbon monoxide
GH: General health
HRQoL: Health-related quality of life
ICU: Intensive care unit
IQR: Interquartile range
MCS: Mental component summary scales
MERS: Middle East Respiratory Syndrome
MERS-CoV: Middle East Respiratory Syndrome coronavirus
MH: Mental health
MOH: Ministry of Health
PF: Physical functioning
PSC: Physical component summary scales
RE: Role limitation due to emotional problems
RP: Role limitation due to physical problems
rRT-PCR: Real-time reverse transcription polymerase chain reaction
SF: Social functioning
SF-36: The Short-Form General Health Survey 36
VT: Vitality (VT)
